# Intimate partner violence and associated factors among women during the COVID-19 pandemic in Ethiopia: a systematic review and meta-analysis

**DOI:** 10.3389/fgwh.2024.1425176

**Published:** 2024-08-23

**Authors:** Tewodros Getaneh Alemu, Tadesse Tarik Tamir, Belayneh Shetie Workneh, Enyew Getaneh Mekonen, Mohammed Seid Ali, Alebachew Ferede Zegeye, Mulugeta Wassie, Alemneh Tadesse Kassie, Berhan Tekeba, Almaz Tefera Gonete, Masresha Asmare Techane

**Affiliations:** ^1^ Department of Pediatrics and Child Health Nursing, School of Nursing, College of Medicine and Health Sciences, University of Gondar, Gondar, Ethiopia; ^2^ Department of Emergency and Critical Care Nursing, School of Nursing, College of Medicine and Health Sciences, University of Gondar, Gondar, Ethiopia; ^3^ Department of Surgical Nursing, School of Nursing, College of Medicine and Health Sciences, University of Gondar, Gondar, Ethiopia; ^4^ Department of Medical Nursing, School of Nursing, College of Medicine and Health Sciences, University of Gondar, Gondar, Ethiopia; ^5^ School of Nursing, College of Medicine and Health Sciences, University of Gondar, Gondar, Ethiopia; ^6^ Department of Clinical Midwifery, School of Midwifery, College of Medicine and Health Sciences, University of Gondar, Gondar, Ethiopia

**Keywords:** intimate partner, violence, factors, women, COVID-19 pandemic, Ethiopia

## Abstract

**Background:**

During the Coronavirus Disease 2019 (COVID-19) pandemic, intimate partner violence increased globally, but most notably in Africa. Conditions such as movement restrictions, staying home, and school closures increased the risk of domestic violence against women. Intimate partner violence is violence demonstrated by an intimate partner against women including physical, sexual, and psychological violence. Despite existing laws against intimate partner violence in Ethiopia, enforcement by law and the judicial system remains inadequate. Thus, this research aims to identify factors contributing to intimate partner violence among women during the COVID-19 pandemic, drawing insights from the current literature.

**Method:**

We searched electronic databases, including PubMed, Google Scholar, CINAHL, Cochrane, and others. Two reviewers separately carried out the search, study selection, critical appraisal, and data extraction. A third party was involved in resolving disagreements among the reviewers. All 10 studies included in this study were published in English, with publication dates before 25 February 2024. Articles lacking an abstract and/or full-text, studies that did not identify the intended outcome, and qualitative studies were excluded from the analysis. A Microsoft Excel checklist was used to extract the data, which were then exported to STATA 11. *I*^2^, funnel plots, and Egger's test were employed to measure heterogeneity and detect publication bias, respectively. A random-effects model was used to estimate the pooled prevalence of intimate partner violence and associated factors among women during the COVID-19 pandemic.

**Result:**

The meta-analysis includes a sample size of 6,280 women from 10 articles. The pooled prevalence of intimate partner violence and associated factors among women during the COVID-19 pandemic was found to be 31.60% (95% CI: 21.10–42.11) and significant factors were partner alcohol use with a pooled odds ratio of 1.93 (95% CI: 1.60–2.23), income loss during the COVID-19 pandemic with a pooled odds ratio of 9.86 (95% CI: 6.35–15.70), partner’s literacy level/education status with a pooled odds ratio of 2.03 (95% CI: 1.57–2.63), and decision-making in the household with a pooled odds ratio of 1.82 (95% CI: 1.33–2.50).

**Conclusion:**

This systematic review and meta-analysis found preliminary evidence that intimate partner violence increased during the COVID-19 pandemic. A partner who has a history of alcohol use, women who had lost income during COVID-19, a partner who has no formal education, and household decisions made by the husband alone were statistically significant factors for intimate partner violence during the COVID-19 pandemic. This implies that the health sector must play a significant role in providing women who are victims of violence with comprehensive healthcare, advocating that violence against women should be viewed as unacceptable, and improving literacy to minimize the consequences of intimate partner violence among women.

## Introduction

The Coronavirus Disease 2019 (COVID-19) outbreak was first detected in Wuhan City, Hubei Province, China (1) and was proclaimed a global pandemic by the World Health Organization (WHO) on 11 March 2020 (2) because of the virus's high severity and ease of transmission, which resulted in an estimated 7 million infections and almost half a million deaths (3). As a result of this, nations all over the globe implemented various preventative actions to assist in stopping the spread. These measures included limiting population movement, isolating people at home, and closing schools as well as other social services, some of which have been demonstrated to increase the likelihood of women being victims of domestic violence (4). Intimate partner violence (IPV) is defined as “violence demonstrated by an intimate partner against women including physical, sexual, and psychological violence or controlling behaviors that cause physical, sexual, or psychological harm” (5). Intentional use of physical force is considered “physical violence,” while forcing a woman to engage in a sexual act is referred to as “sexual violence” (6). “Emotional,” “psychological,” or “verbal” violence refers to acts of intimidation, control over activities, isolation, name-calling, and threats (7). During the COVID-19 pandemic, IPV increased globally, but most notably in Africa (8). In their lives, 35% of women globally have either been victims of physical or sexual abuse at the hands of an intimate partner or of non-partner sexual violence (9). Approximately 27% of women between the ages of 15 and 49 who have been in a relationship worldwide claim that their intimate partner has physically or sexually abused them (10). The prevalence was much greater in sub-Saharan Africa, where 46.5% of women had been the victim of intimate partner violence (8). Situations such as being confined to one's house, moving limitations, and closing schools raise the possibility of domestic violence against women (11, 12). IPV has been linked to long-term problems with mental, physical, and reproductive health (13–15). Women who experience IPV also have the risk of conflicts with others and developing social disorders (16, 17). During the COVID-19 pandemic restrictions, the majority of IPV victims were women whose spouses had financial and behavioral control, as well as women who had partners who consumed alcohol (18). In Ethiopia, in particular, similar measures were taken to reduce the COVID-19 pandemic's spread. These measures included closing educational institutions, workplaces, and recreational facilities; restricting travel; setting up isolation and quarantine facilities; and declaring a national emergency (19, 20). However, the federal government did not respond to any of the consequences of these preventative actions, such as IPV against women (21). IPV was more common during the COVID-19 period, even though Target 5.2 of the Sustainable Development Goals (SDG) 5 is intended to eradicate all kinds of violence against all women by 2030 (22). While some studies have reported determinants of intimate partner violence and associated factors among women during the COVID-19 pandemic in Ethiopia, none of them have systematically reviewed intimate partner violence and associated factors among women during the COVID-19 pandemic. These differ and are not uniform throughout the region. To end violence against women, public health stakeholders need to be aware of the impact of the COVID-19 pandemic on intimate partner violence as well as the most up-to-date information on the prevalence of IPV. The reported determinants include economically disadvantaged women, women of a young age, non-educated women, women whose partners control behavior, women who have partners who drink alcohol and/or use substances, and women who spend more time at home (23–32). Thus, the current work aims to identify relevant studies and summarize major determinants of intimate partner violence and associated factors among women during the COVID-19 pandemic in Ethiopia. Furthermore, the review's findings will broaden our understanding of the issue and provide crucial information to planners of programs, legislators, and other interested parties who want to see an end to violence against women in Ethiopia.

## Methods

### Searching strategy and data source

Following the criteria of the Preferred Reporting Items for Systematic Reviews and Meta-Analysis (PRISMA), a systematic review of relevant papers was carried out (33). A thorough search of all English-language publications released before 25 February 2024 was conducted using PubMed, Google, Google Scholar, Cochrane Library, and CINAHL. In addition, we searched the reference lists of the included studies in addition to these databases to find possible literature. The search was performed using key terms such as intimate partner violence, violence against girls, perinatal intimate partner violence, reproductive age women, pregnant women, postpartum mothers, married women, prenatal clients, during COVID-19 pandemic, impact of the COVID-19 pandemic, associated factors, risk factors, prevalence, predictors, determinants, and Ethiopia separately and/or in combination using the Boolean operators “OR” or “AND.” Two of the authors searched independently. To make the process of selecting articles and managing citations easier, articles that were extracted from the databases were imported into Endnote version X9.

### Study selection and eligibility criteria

Studies that were conducted in Ethiopia with the main goal of examining intimate partner violence and related factors were included in this review and meta-analysis. Studies published in English, conducted at both facility and community levels, and related to reproductive age, pregnant women, postpartum mothers, perinatal clients, and married women were included. The titles, abstracts, and full review of the research were used to evaluate the studies for inclusion criteria prior to their inclusion in the final review. Where the required data were not available (articles lacking abstract and/or full-text, the study that did not meet the inclusion criteria, studies that did not identify the intended outcome), the study was excluded from the review.

### Outcome measure

The primary outcome of this review was the prevalence of intimate partner violence among women during the COVID-19 pandemic (the response to each item was either “Yes” or “No” for any form of physical, sexual, and emotional violence against women by an intimate partner) (5). An intimate partner is a person who has an intimate relationship with a woman either in the form of marriage or in the form of cohabitation (34). The factors linked to intimate partner violence among women during the COVID-19 pandemic were the second outcome of this review. A factor was included in this review and meta-analysis if it was found to be linked with intimate partner violence among women during the COVID-19 pandemic in two or more research articles; however, if it was found to be connected with intimate partner violence in just one study, it was not taken into consideration. The age of the woman, residence, income loss during COVID-19, partner alcohol use, partner substance use, marital status, women's education, partner education, decision-making in the household, age of the husband, partner smoking habit, women's occupation, and history of abortion were the exposure variables included.

### Quality assessment and data extraction

Two reviewers independently assessed the quality of the studies by adopting a specific protocol. The criteria proposed by the Joanna Briggs Institute (JBI) were used to assess the quality of the included studies (35). The following eight parameters were assessed: inclusion criteria, study subject and setting description, valid and reliable exposure measurement, objective and standard criteria applied, confounder identification, confounder handling strategies, outcome measurement, and appropriate statistical analysis. When a study achieved a quality assessment indicator score between 75% and 100%, it was categorized as high quality; scores between 50% and 74% indicated moderate quality, and scores between 0% and 49% indicated low quality. Therefore, if a study achieved a quality assessment indicator score of 50% or more, it was categorized as low risk (Table 1). A third reviewer was involved in the discussion process to resolve any discrepancies between the two reviewers. The names of the author(s), the year of publication, the study period, the study design, the sample size, the prevalence of intimate partner violence, and related factors were all gathered using a predetermined data extraction format.

**Table 1:** Characteristics and quality status of the studies included to assess the pooled prevalence of intimate partner violence among women during the COVID-19 pandemic in Ethiopia.

First author	Year of publication	Study population	Sample size	Outcome	Prevalence
Alemayehu Sayih Belay	2021	Postpartum mothers	657	410	62.4
Shannon N. Wood	2022	Pregnant women	983	149	15.6
Gossa Fetene	2021	Pregnant women	590	232	39.3
Mekasha Getnet Demeke	2021	Reproductive age women	796	337	42.3
Gebremeskel Tukue Gebrewahd	2020	Reproductive age women	682	168	24.6
Solomon Shitu	2020	Reproductive age women	448	189	42.2
Abayneh Shewangzaw Engda	2022	Reproductive age women	700	133	19
Abel Teshome	2020	Prenatal women	464	33	7.1
Ayenew Kassie	2022	Reproductive age women	371	156	42
Abay Woday Tadesse	2022	Married women	589	132	22

### Publication bias and heterogeneity

Funnel plots were scattered and their asymmetry was checked to determine whether publication bias existed. The Egger's test was estimated (36). The statistical significance of publication bias was determined via a *p*-value of <0.05. *I*^2^ test statistics were used to verify the heterogeneity of the studies following the authors' thorough review of each one. The overall variation across studies was described by *I*^2^ statistics. *I*^2^ test statistics of <50%, 50%–75%, and >75% were designated as low, moderate, and high heterogeneity, respectively (37).

### Statistical methods and analysis

Statistical analysis was carried out using STATA version 11. Initially, data were entered into Microsoft Excel and then exported to STATA version 11 for further analysis. The effect size of the meta-analysis was the prevalence of intimate partner violence and the odds ratio (OR) of the associated factors. A weighted inverse variance random-effects model was used as a method of analysis (38). By examining the adjusted ORs and 95% confidence intervals (CIs) provided by each study, we were able to determine associated risk factors for intimate partner violence among women who satisfied the eligibility requirements for the meta-analysis. The study population was used to do subgroup analysis. The effect of selected associated factors was analyzed using separate categories of meta-analysis. The findings of the review and meta-analysis were presented using tables, forest plots, OR, and 95% CIs.

## Results

### Study searches and selection

**Figure 1:**
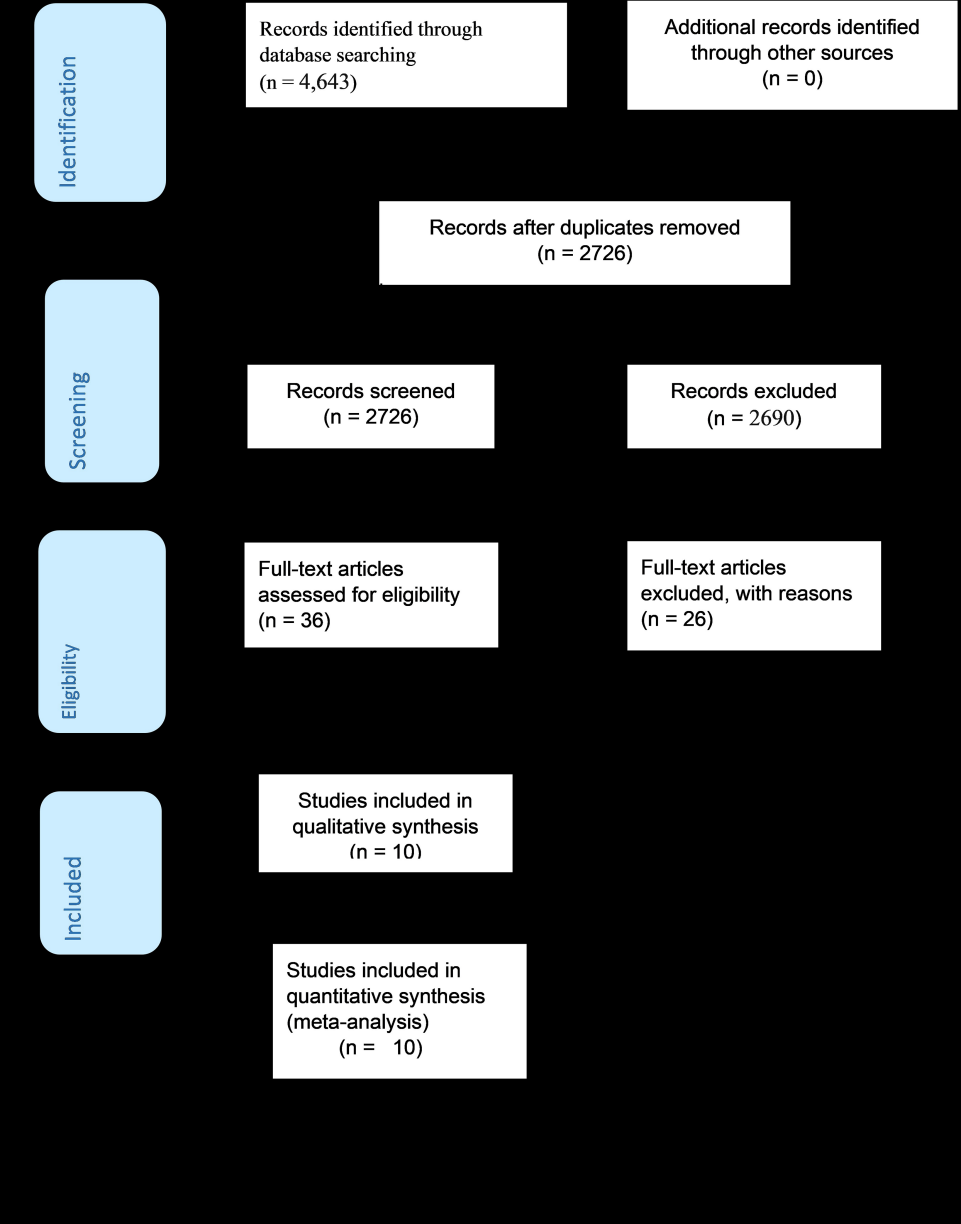
A PRISMA flow diagram of articles screening and process of selection.

In the initial search, we found a total of 4,643 records from different electronic search databases which included PubMed (11), Google Scholar (4,618), and the Cochrane Library (14). From these, 1,917 duplicate records were removed and 2,690 records were excluded after screening by title and abstracts. After determining the eligibility of the 36 remaining records based on their full texts, 26 records were eliminated based on the inclusion and exclusion criteria. Finally, 10 studies were considered for the final review and meta-analysis (23–30, 31, 32) (Figure 1).

### Characteristics of the studies

Every study that was part of this review was cross-sectional in nature. The analysis had a total of 6,280 women participants. The included studies reported sample sizes ranging from 371 (27) to 983 (31). Of the women in the included study, 50% were of reproductive age, 20% were pregnant, and the remaining women were postpartum mothers, perinatal clients, or married women. One study was conducted at the national level, two more were carried out in central Ethiopia, one study was carried out in eastern Ethiopia, three more were carried out in northern Ethiopia, and the final three were carried out in southern Ethiopia. Intimate partner violence was found to be most common in southern Ethiopia (62.4%) and least common in central Ethiopia (Addis Ababa) (7.1%) (Table 1).

### Prevalence of intimate partner violence among women during the COVID-19 pandemic

**Figure 2:**
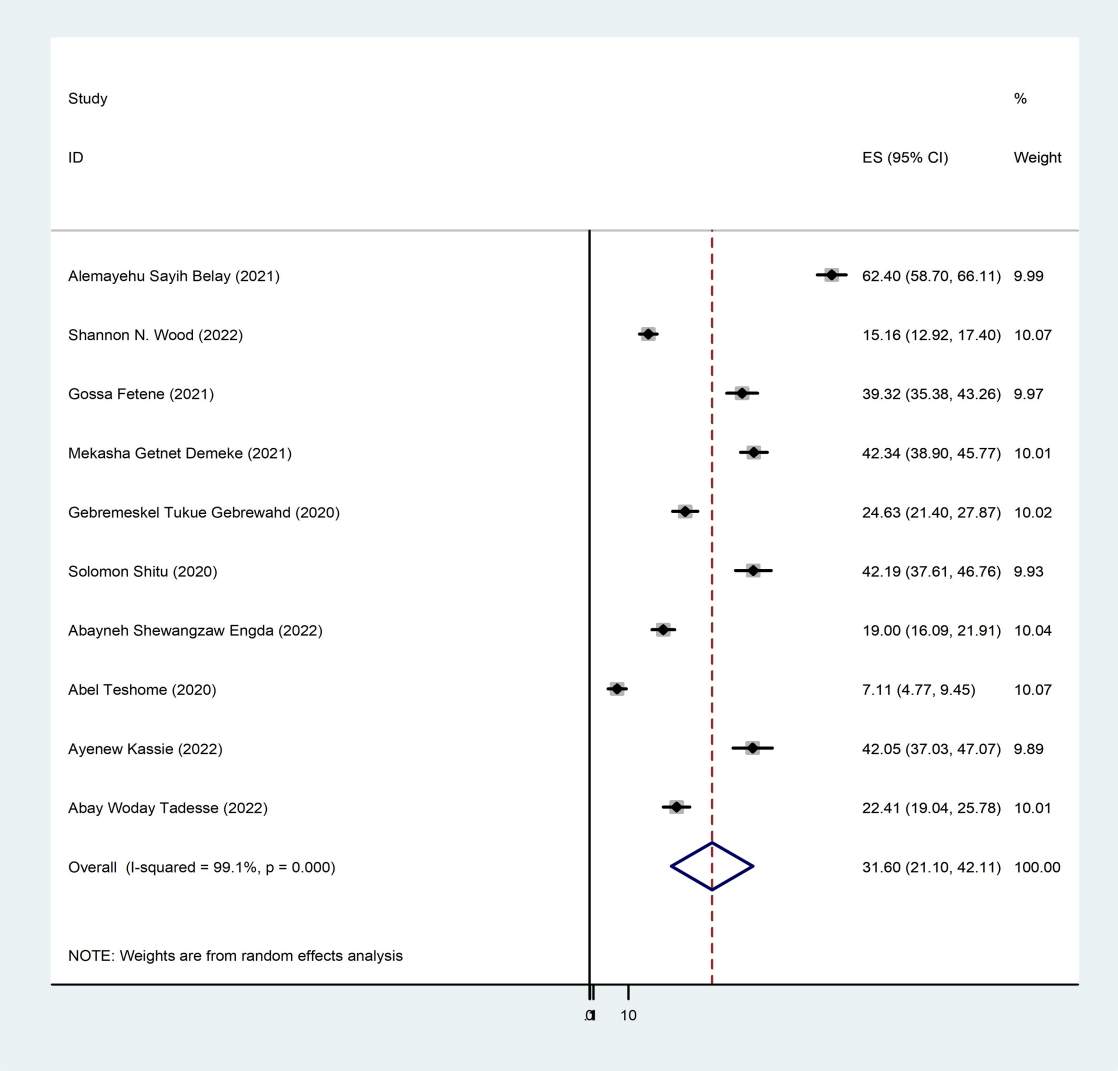
Forest plot pooled prevalence of intimate partner violence among women during the COVID-19 pandemic in Ethiopia, 2024.

**Figure 3:**
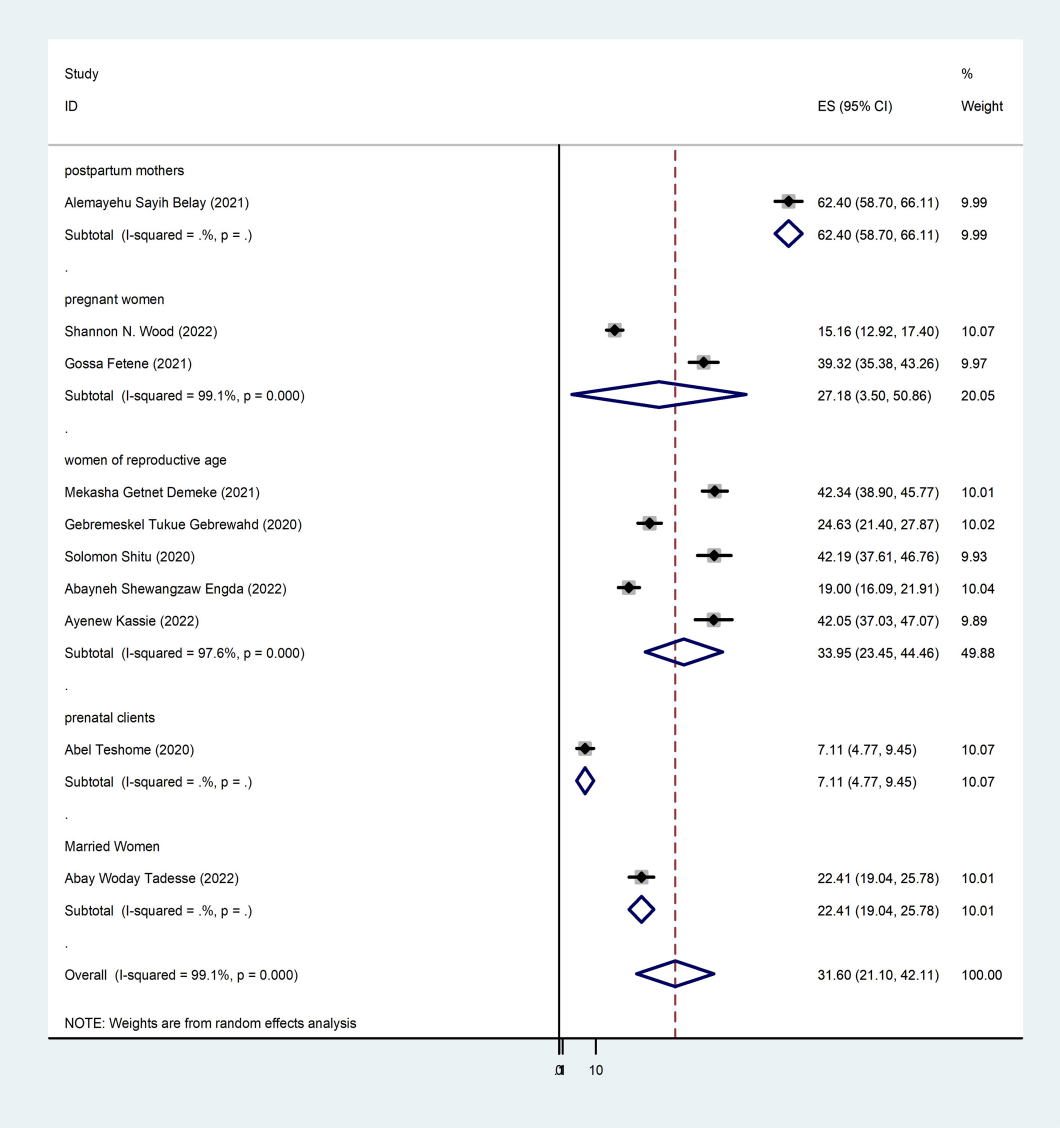
Forest plot subgroup prevalence of intimate partner violence among women during the COVID-19 pandemic in Ethiopia, 2024.

To determine the pooled prevalence of intimate partner violence among women during the COVID-19 pandemic, 10 studies were included in the analysis. The 10 studies that were utilized to estimate the pooled prevalence of intimate partner violence among women during the COVID-19 pandemic showed a very high level of heterogeneity (*p* < 0.000 and *I*^2^ = 99.1%). Using the random-effects model, the pooled prevalence of intimate partner violence among women during the COVID-19 pandemic was 31.60% (95% CI: 21.10–42.11) (Figure 2). Based on participant characteristics, a subgroup analysis was conducted to compare the prevalence of intimate partner violence among women during the COVID-19 pandemic. Thus, the pooled prevalence estimated for characteristics was high in postpartum mothers [62.40% (95% CI: 58.70–66.11)], and the least was in perinatal women [7.11% (95% CI: 4.77–9.45)] (Figure 3).

### Publication bias and sensitivity analysis

**Figure 4:**
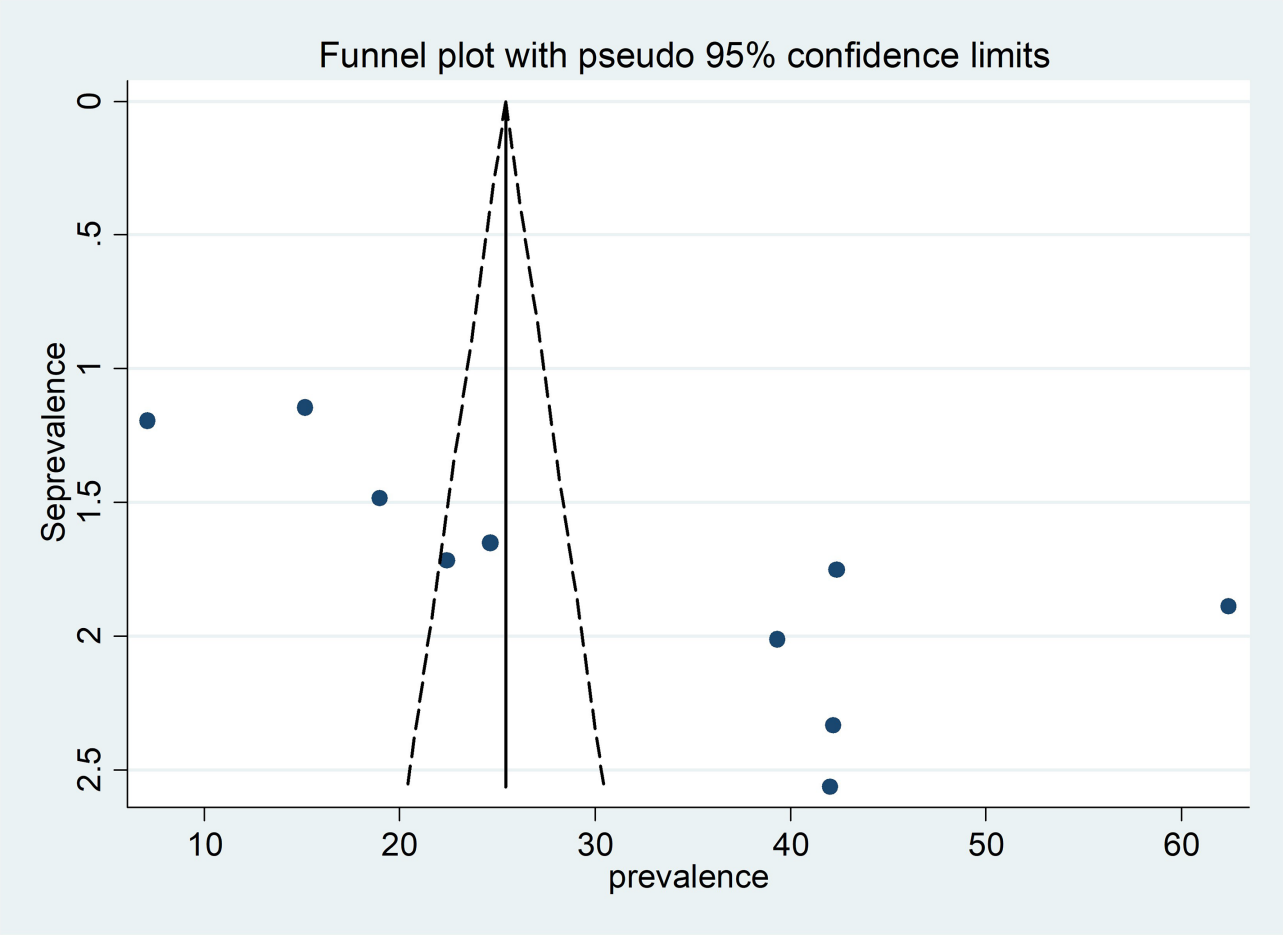
Funnel plot for publication bias, Log prop or LNP (log of proportion) represented in the x-axis and standard error of log proportion in the y-axis.

Egger's regression test and a funnel plot were used to assess publication bias. Subjectively, a funnel plot with an uneven distribution suggests the existence of publishing bias (Figure 4). Furthermore, Egger's regression test's objective *p*-value of 0.006 suggested the existence of publication bias.

**Figure 5:**
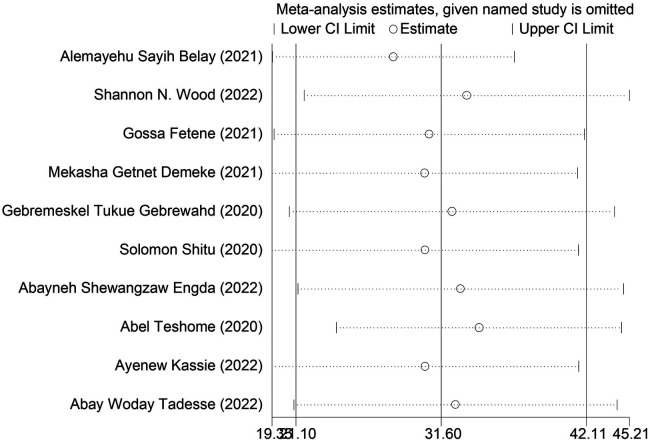
Sensitivity analysis of the included studies.

We conducted a sensitivity analysis to ascertain the weight of each study in relation to the total effect size of the prevalence of intimate partner violence and associated characteristics among women during the COVID-19 pandemic. The DerSimonian–Laird random-effects model sensitivity analysis revealed that no single study had an impact on the overall prevalence of intimate partner violence and associated factors among women during the COVID-19 pandemic in Ethiopia (Figure 5).

### Associated factors of intimate partner violence

**Figure 6:**
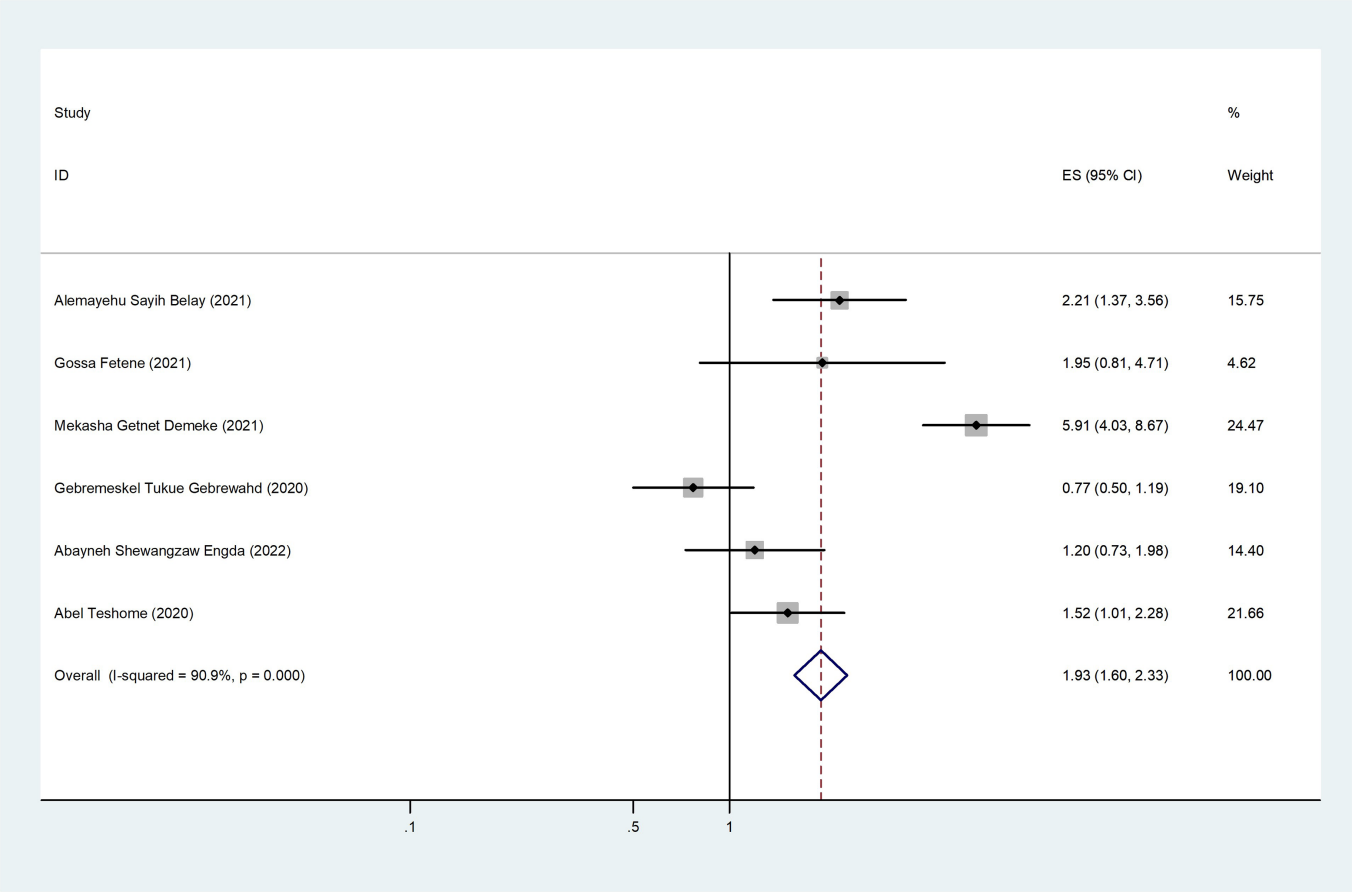
The pooled effect of partner alcohol use and the prevalence of intimate partner violence among women during the COVID-19 pandemic in Ethiopia.

Among the included 10 studies, 6 studies reported the association between partner alcohol use and intimate partner violence. The pooled odds ratio from these studies was 1.93 (95% CI: 1.60–2.23), revealing that a woman with a partner who had a history of alcohol use was two times more likely to be a victim of intimate partner violence than their counterparts (Figure 6).

**Figure 7:**
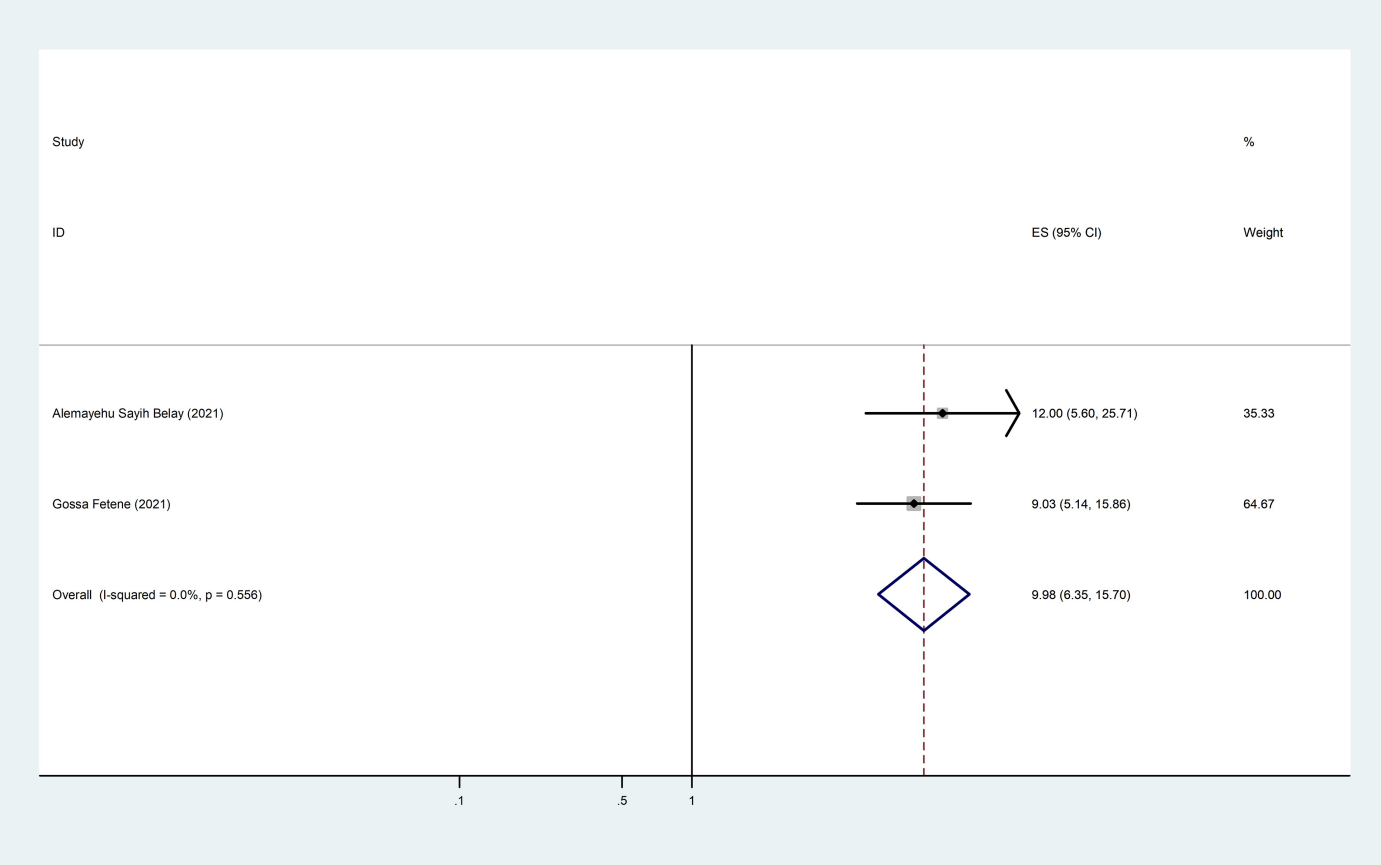
The pooled effect of income loss during the COVID-19 pandemic and the prevalence of intimate partner violence among women during the COVID-19 pandemic in Ethiopia.

Two of the 10 included studies revealed an association between intimate partner violence and income lost during the COVID-19 pandemic. The pooled odds ratio was 9.86 (95% CI: 6.35–15.70), indicating that those who lost income during the COVID-19 pandemic were 10 times more likely to be victimized by intimate partner violence than those who did not lose income during the COVID-19 pandemic (Figure 7).

**Figure 8:**
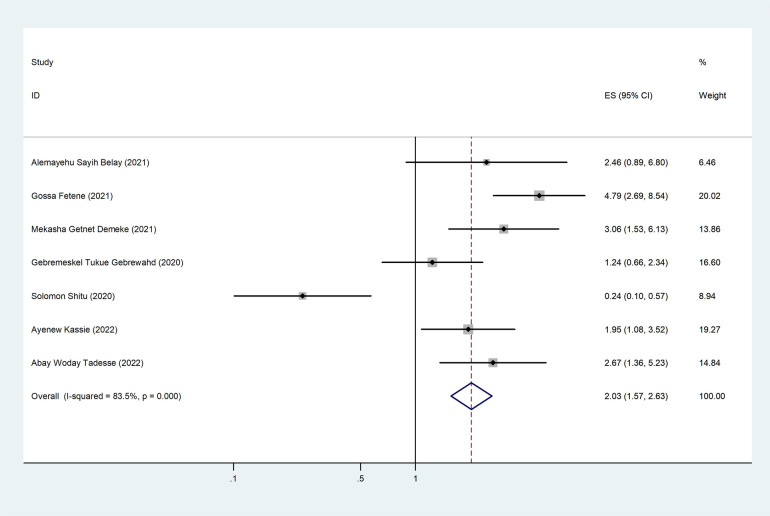
The pooled effect of partner education and the prevalence of intimate partner violence among women during the COVID-19 pandemic in Ethiopia.

Of the 10 studies that were considered, 7 of them showed an association between intimate partner violence and partner education. The pooled odds ratio was 2.03 (95% CI: 1.57–2.63), showing that partners who had no formal education are two times more likely to commit violence than partners who had formal education (Figure 8).

**Figure 9:**
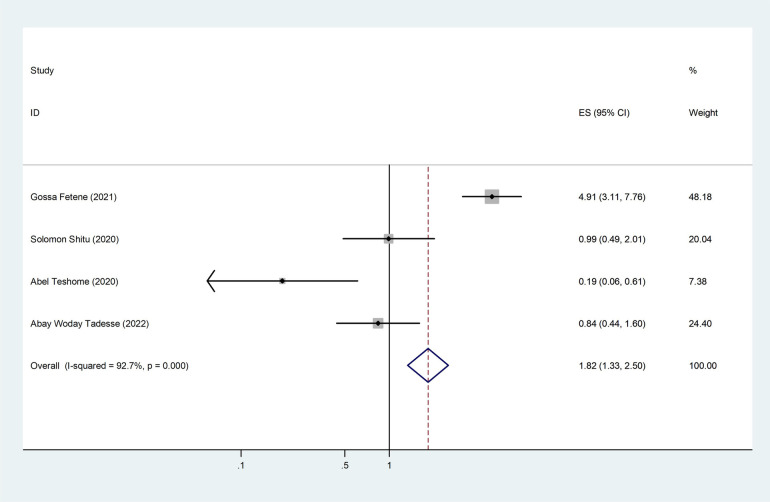
The pooled effect of decision-making in the household and the prevalence of intimate partner violence among women during the COVID-19 pandemic in Ethiopia.

Four of the seven included studies revealed an association between intimate partner violence and decision-making in the household. The pooled odds ratio was 1.82 (95% CI: 1.33–2.50), indicating that those women who had decisions made by their partner only in the household were 1.82 times more likely to experience intimate partner violence than their counterparts (Figure 9).

## Discussion

This review was conducted to estimate the pooled prevalence and associated factors of intimate partner violence against reproductive age women during the COVID-19 pandemic in Ethiopia.

During the COVID-19 pandemic, women's pooled prevalence of any form of IPV was 31.60% (95% CI: 21.10–42.11). This prevalence was comparable to a systematic review performed globally (31%) (39) and in Europe (21%) (40). However, the pooled prevalence was higher than that in studies performed before the pandemic: Southern Asia (19%) (41), Western Asia (13%) (42), France (7%) (43), North Africa (15%) (44), and sub-Saharan Africa (20%) (45). This may be due to the stay-at-home or lockdown policy because of the COVID-19 pandemic; long periods of time spent together at home exacerbate the situation and raise the risk of violence. In addition, the lockdown has an impact on many facets of society, including socioeconomic circumstances that can drive interpersonal conflict and violent acts. Our findings were lower than those of a study conducted in Bangladesh (45.29%) (46), Peru (48.0%) (47), and Iran (65.4%) (48). This may be due to the sociodemographic characteristics of the countries, study design, and sample size difference.

Intimate partner violence is a serious public health issue and a violation of women's human rights (10). Violence may raise the chance of human immunodeficiency virus (HIV) infection in specific situations and have a detrimental impact on women's sexual, emotional, physical, and reproductive health (10).

This study found partner alcohol use was a significant determinant of intimate partner violence. In this regard, we found that a partner who had a history of alcohol use had a higher likelihood of committing violence than their counterparts. This is consistent with studies conducted in different countries (49, 50). This may be explained by the fact that drinking alcohol might impair a person's ability to think clearly and cognitively. As a result, women who have alcohol-using spouses are more likely to experience violence than women who do not. This demonstrates the significance for the government and organizations that promote women's health to pay attention to alcohol-related activities in the nation.

Women who lost income during the COVID-19 pandemic were nearly 10 times more likely to be targeted by intimate partner violence than those who did not lose income during the COVID-19 pandemic. This finding is consistent with systematic reviews done on intimate partner violence in different regions of the globe (51, 52). This could be due to staying at home being one of the COVID-19 pandemic preventive and control strategies that led to a woman's income loss, which increases her risk of IPV. Furthermore, this could be because women who rely on their male partners for financial support are more vulnerable to IPV. Working on the rehabilitation of women who lost their income due to the COVID-19 pandemic is an excellent strategy since it may reduce the number of women who experience violence from their partners and improve the health of women.

Educational status was much associated with intimate partner violence. Partners who had no formal education were two times more likely to commit violence than partners who had formal education. This result is consistent with different studies (53–55). This might be because education influences people's perspectives in various ways and because educated individuals communicate more effectively than their less-educated counterparts. As a result, they believe that problems can be resolved via dialogue rather than by violating the rights of others. In addition, due to their lack of understanding of their wives’ and partners’ legal rights, illiterate husbands may not be aware of the implications of their violent behavior, be unable to establish flexible and caring behavior, and view violence against women as the norm.

Moreover, this systematic review and meta-analysis observed that partners who made household decisions alone were 1.82 times more likely to commit intimate partner violence than their counterparts. This could be due to the fact that a woman who lacks decision-making authority in the household is indicative of a relationship that is unhealthy and violent, and women are not comfortable discussing household issues with their partners. Improving women's decision-making in the home is crucial since it may reduce the likelihood that their partners may use violence against them.

This study highlights the urgent need for enhanced enforcement of IPV laws and support systems. The findings also serve as a foundation for advocating women's justice in Ethiopia and other similar contexts. In addition, future research should explore longitudinal changes in IPV rates and expand on the identified risk factors to better understand and address IPV dynamics.

## Limitation

There are certain limitations to the current work. The study's reliance on cross-sectional studies conducted during the pandemic period may not capture the longitudinal effects of the COVID-19 pandemic on IPV trends, and that only 10 articles were reviewed for this study may limit the generalizability of the results. Furthermore recall bias, response bias, and differential misclassification within the reviewed studies are potential biases. Furthermore, the study's inability to compare IPV rates before and during the pandemic makes it difficult to draw firm conclusions on an increase in IPV. However, the study also has strengths such as assessments for heterogeneity, publication bias, and sensitivity analyses, ensuring robust and consistent findings. As a result, this will enhance the transparency and credibility of the study. Correspondingly, it has novelty since this is the first systematic review and meta-analysis focusing on IPV in Ethiopia during the COVID-19 pandemic.

## Conclusion

This systematic review and meta-analysis found preliminary evidence that IPV increased during the COVID-19 pandemic. This finding also identified several key determinants of intimate partner violence during the COVID-19 pandemic. Based on the findings of this review, a partner who has a history of alcohol use, women who lost income during COVID-19, a partner who has no formal education, and household decisions made by the husband alone were statistically significant factors for intimate partner violence during the COVID-19 pandemic. Hence, this implies that the health sector must play a significant role in providing women who are victims of violence with comprehensive healthcare, advocating that violence against women should be viewed as unacceptable, and improving literacy to minimize the consequences of intimate partner violence among women. Moreover, to comprehend and examine IPV dynamics more deeply, future research should also look into longitudinal changes in IPV rates and elaborate on the risk variables that have been found.
